# Production of marker-free transgenic *Jatropha curcas* expressing hybrid *Bacillus thuringiensis δ*-endotoxin Cry1Ab/1Ac for resistance to larvae of tortrix moth (*Archips micaceanus*)

**DOI:** 10.1186/1754-6834-7-68

**Published:** 2014-05-03

**Authors:** Keyu Gu, Huizhu Mao, Zhongchao Yin

**Affiliations:** 1 Temasek Life Sciences Laboratory, 1 Research Link, National University of Singapore, Singapore 117604, Republic of Singapore; 2 Department of Biological Sciences, 14 Science Drive, National University of Singapore, Singapore 117543, Republic of Singapore

**Keywords:** *Jatropha curcas*, *Archips micaceanus*, *Bacillus thuringiensis*, Cry1Ab/1Ac, marker-free transformation

## Abstract

**Background:**

The potential biofuel plant *Jatropha curcas* L. is affected by larvae of *Archips micaceanus* (Walker), a moth of the family Tortricidae. The hybrid *Bacillus thuringiensis* (*Bt*) *δ*-endotoxin protein Cry1Ab/1Ac confers resistance to lepidopteran insects in transgenic rice.

**Results:**

Here, we report the production of a marker-free transgenic line of *J. curcas* (L10) expressing Cry1Ab/1Ac using *Agrobacterium*-mediated transformation and a chemically regulated, Cre/*loxP*-mediated DNA recombination system. L10 carries a single copy of marker-free T-DNA that contains the *Cry1Ab/1Ac* gene under the control of a maize phosphoenolpyruvate carboxylase gene promoter (*P*_*Pepc*_*:Cry1Ab/1Ac:T*_*Nos*_). The *P*_*Pepc*_*:Cry1Ab/1Ac:T*_*Nos*_ gene was highly expressed in leaves of L10 plants. Insecticidal bioassays using leaf explants of L10 resulted in 80-100% mortality of larvae of *A. micaceanus* at 4 days after infestation.

**Conclusion:**

The results demonstrate that the hybrid *Bt δ*-endotoxin protein Cry1Ab/1Ac expressed in *Jatropha curcas* displays strong insecticidal activity to *A. micaceanus*. The marker-free transgenic *J. curcas* line L10 can be used for breeding of insect resistance to *A. micaceanus*.

## Background

The crystalline (Cry) proteins from *Bacillus thuringiensis* (*Bt*) have specifically toxic activity against numerous insect species of the orders Lepidoptera, Diptera, Coleoptera, Hymenoptera and nematodes [1]. The Cry proteins are inactive until they get solubilized by proteases in the insect’s midgut at high pH (>9.5), releasing proteins called *δ*-endotoxins [2,3]. The *δ*-endotoxins bind to the midgut receptors, insert into the insect gut cell membrane to form ion channels or pore and cause cellular lysis due to the inflow of ions and water through the pores, which eventually kills the insects [4-6]. The insecticidal activity of the Cry proteins provides an alternative and attractive approach for pest management through the expression of Cry proteins in transgenic plants. Expressions of the *cry* gene in tobacco and tomato are the first two reports of plants genetically engineered for insect resistance [7,8]. Since that, different versions of *cry* genes have been used to generate transgenic crops, such as corn, cotton, potato, tomato, rice and sugarcane [9]. The first *Bt*-derived insect-resistant transgenic tree was the transgenic poplar (*Populus sp.*) expressing Cry1A(a) *δ*-endotoxin, which provided resistance against forest tent caterpillar (*Malacosoma disstria*) and gypsy moth (*Lymantria dispar*) [10,11]. Since that, different versions of *cry* genes have been transferred into a number of tree species, including walnut (*Juglans regia*) [12], European larch (*Larix decidua* Mill.) [13], white spruce (*Picea glauca*) [14,15], loblolly pine (*Pinus taeda* L.) [16], eucalyptus (*Eucalyptus camaldulensis*) [17] and hybrid poplar (*Populus tremula* × *Populus tremuloides*) [18].

*Jatropha curcas* L. is a poisonous, semi-evergreen shrub or small tree that belongs to *Euphorbiaceae* family. *J. curcas* mainly grows in tropical and subtropical countries. Compared with other plants, *J. curcas* is a drought-resistant, non-food plant that can grow in marginal lands. *J. curcas* seeds contain about 25 to 40% storage lipids [19]. In recent years, *J. curcas* has emerged as a potential biofuel plant. However, like other crops, large plantation of *J. curcas* is greatly influenced by biotic and abiotic stresses. Despite the presence of toxins such as phorbol ester and curcins in *J. curcas* leaves and seeds, *J. curcas* is still attacked by insects [20-24], fungi [25] and viruses [26].

**Figure 1:**
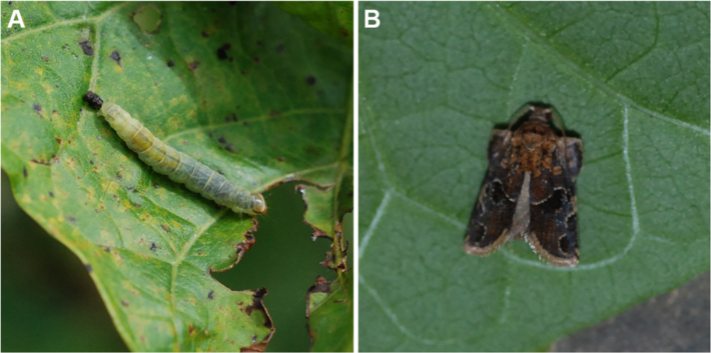
***Archips micaceanus *** **at larva (A) and adult (B) stages on *J. curcas *** **leaves.**

*Archips* is a genus of tortrix moths that belong to the family Tortricidae and have over 100 species. The leafrolling larvae of tortrix moths feed on plant leaves, causing damage to crops and trees [27]. Recently, *Archips occidentalis* (Walsingham) was reported to cause damage on *J. curcas* plants in Southern Benin [20]. In Southeast Asia, the most common tortrix moth species is *Archips micaceanus* (Walker) although *Archips machlopis* (Meyrick) and *Archips tabescens* (Meyrick) were also found in Malaysia [28]. *A. micaceanus*, also called soybean leaf-roller, is the only tortrix moth species reported in Singapore [28]. Recently, we found that *A. micaceanus* could also cause damage to *J. curcas* (Figure 1). Chemical pesticides are effective against the tortrix moths and larvae; however, they also kill non-target beneficial insects, especially the pollinators for *J. curcas*[29]. Previously, *Bt*-derived biological insecticides were used to control tortrix moths *Archips argyrospilus* (Walker) on apple and pear [30,31] and *Archips rosanus* (L.) on filberts [32]. The effectiveness of *Bt* on the two tortrix moths suggests *cry* gene may be used to control *A. micaceanus* on *J. curcas* plants.

A hybrid *cry* gene *Cry1Ab/1Ac* was previously used to generate transgenic rice in Minghui 63, an indica cytoplasmic male sterile (CMS) restorer line, and its derived hybrid F_1_ rice Shanyou 63 expressing Cry1Ab/1Ac proteins showed high protection against both leaf-folder (*Cnaphalocrocis medinalis*) and yellow stem borer (*Scirpophaga incertulas*) [33]. Recently we produced a marker-free transgenic rice line L24 with the *P*_*Pepc*_*:Cry1Ab/1Ac:T*_*Nos*_ gene using *Agrobacterium*-mediated transformation and a chemically regulated, Cre/*loxP*-mediated DNA recombination system [34]. The *P*_*Pepc*_*:Cry1Ab/1Ac:T*_*Nos*_ gene in L24 was mainly expressed in green tissues such as leaves and stem and provided resistance to rice leaf-folder (*C. medinalis*) [34]. Here, we report the adoption of similar technology to generate a marker-free transgenic line of *J. curcas* that expresses Cry1Ab/1Ac proteins for resistance to *A. micaceanus*.

## Results

### Generation of marker-free transgenic line of *J. curcas* containing *P*_*Pepc*_*:Cry1Ab/1Ac:T*_*Nos*_ gene

The binary construct pCCreloxPBt, which carries a chemically regulated Cre/*loxP* system and a hybrid *Cry1Ab/1Ac* gene driven by maize phosphoenolpyruvate carboxylase (PEPC) gene promoter (*P*_*Pepc*_*:Cry1Ab/1Ac:T*_*Nos*_), was used to produce marker-free transgenic rice line L24 that specifically expresses the Cry1Ab/1Ac proteins in leaves and stem [34]. Theoretically, the marker (*Hpt* gene)-containing *loxP* fragment in T-DNA region of pCCreloxPBt in transgenic plants can be removed by *β*-estradiol-regulated Cre/*loxP*-mediated excision, which yields marker-free T-DNA in transgenic plants [34]. The marker-free T-DNA is detected by PCR amplification of P1-P4 fragment (385 bp) flanking the remaining *loxP* site after DNA recombination, while the marker-containing T-DNAs, T-DNAs that have undergone incomplete *loxP* fragment excision and truncated T-DNAs are detected by PCR amplification of the P1-P2 (534 bp) fragment flanking the *loxP* site at the left border of T-DNA and/or the P3-P4 (460 bp) fragment flanking the *loxP* site adjacent to the maize PEPC gene promoter [34]. In this study, pCCreloxPBt was used to produce transgenic *J. curcas* plants via *Agrobacterium*-mediated transformation [35]. After *β*-estradiol-regulated Cre/*loxP*-mediated excision of the *loxP* fragment, a total of twenty putative transgenic T_0_ plants were obtained. The 20 T_0_ plants were screened by PCR for the presence of the *Cry1Ab/1Ac* and *Hpt* genes as well as for the excision of the *loxP* fragment, which was detected by the amplification of P1-P4 fragment [34]. All the 20 T_0_ plants carried the coding region of the *Cry1Ab/1Ac* gene (Table 1). Only one plant, T_0_-20, showed PCR amplification of the *Cry1Ab/1Ac* gene and the P1-P4 fragment, but not the *Hpt* gene (Table 1). Twelve T_0_ plants had PCR amplification of both *Cry1Ab/1Ac* and *Hpt* genes, but not the P1-P4 fragment, indicating that they carried marker-containing T-DNA only (Table 1). Six T_0_ plants gave amplification of both *Cry1Ab/1Ac* and *Hpt* genes, as well as the P1-P4 fragment (Table 1). These plants should contain multiple copies of T-DNA and at least one copy had undergone *loxP* fragment excision. In addition, plant T_0_-1 had PCR amplification of the *Cry1Ab/1Ac* gene only, but not the *Hpt* gene or the P1-P4 fragment (Table 1). This plant might carry a truncated T-DNA containing the *Cry1Ab/1Ac* gene only, which did not result from precise excision of the *loxP* fragment.

**Table 1:** **PCR analysis of the T**_**0 **_**transgenic plants**

**T**_**0 **_**plant**	***Cry1Ab/1Ac***^**1**^	**P1-P4**^**2**^	***Hpt***^**3**^
pCCreloxPBt^4^	+	-	+
MD44	-	-	-
1	+	-	-
2	+	-	+
2B	+	-	+
4A	+	-	+
4B	+	-	+
6	+	-	+
8	+	-	+
10	+	+	+
11	+	+	+
12	+	-	+
13	+	-	+
19	+	-	+
20	+	+	-
21	+	+	+
24	+	-	+
33	+	+	+
37	+	+	+
38	+	+	+
39	+	-	+
40	+	-	+

^1^The primers for detecting the presence of the *Cry1Ab/1Ac* gene were Bt F1 and Bt R1; ^2^the primers for detecting the excision of the *lox*P fragment or the presence of P1-P4 fragment were P1 and P4 [34]; ^3^the primers for detecting the presence of the *Hpt* gene were Hpt F and Hpt910-1; ^4^binary construct used for transgenic *J. curcas* production [34].

**Figure 2:**
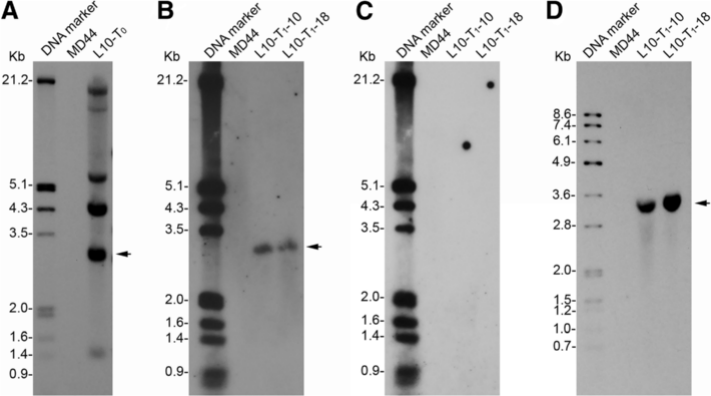
**Southern blot analysis of transgenic *Cry1Ab/1Ac *plants of *J. curcas. *(A)** Detection of T-DNA copy number in transgenic T_0_ plant L10 (L10-T_0_). Genomic DNA samples of L10-T_0_ and cultivar MD44 were digested with restriction enzyme *Nco*I. The southern blot was hybridized with a DNA probe derived from the *Cry1Ab/1Ac* gene. Arrow indicates the T-DNA copy that was putative marker-free (see below), which was selected for further study. **(B)** Transmission of the marker-free T-DNA in the T_1_ progeny of L10. Southern blot analysis was carried out using the restriction enzyme *Nco*I and the *Cry1Ab/1Ac* probe. L10-T_1_-10 and L10-T_1_-18 are two individual T_1_ plants of L10. **(C)** Detection of *Hpt* gene in T_1_ plants L10-T_1_-10 and L10-T_1_-18 by southern blot analysis. The same southern blot in **(B)** was stripped and then re-hybridized with an *Hpt* gene probe. **(D)** Detection of the intact *P*_*Pepc*_*:Cry1Ab/1Ac:T*_*Nos*_ gene in the marker-free T_1_ progeny of L10. Genomic DNA samples of L10-T_1_-10, L10-T_1_-18 and MD44 were digested with restriction enzymes *Kpn*I and *Sph*I. The southern blot was hybridized with the *Cry1Ab/1Ac* probe. The expected size of the *Kpn*I-*Sph*I fragment of the *P*_*Pepc*_*:Cry1Ab/1Ac:T*_*Nos*_ gene is 3452 bp.

**Figure 3:**
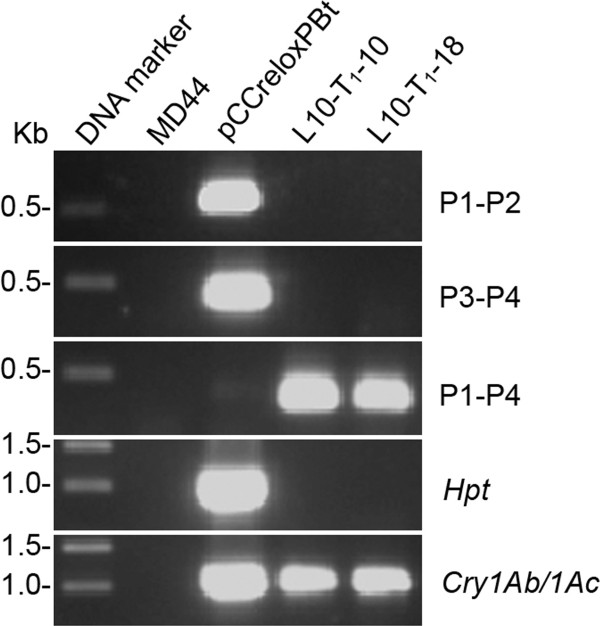
**Detection of *loxP *fragment excision by PCR.** Genomic DNA samples from the putative marker-free T_1_ plants L10-T_1_-10 and L10-T_1_-18 were amplified by PCR for detection of Cre/*loxP*-mediated *loxP* fragment excision using primer pairs P1/P2, P3/P4 and P1/P4 [34]. The presence or absence of the *Hpt* and Cry1Ab/1Ac genes were also detected by PCR. Plasmid DNA of pCCreloxPBt and genomic DNA from MD44 were used as the controls. Primers are listed in Table 4.

The expression of the Cry1Ab/1Ac proteins in the 20 T_0_ plants was detected by western blot analysis. The initial results indicated that only one T_0_ plant, L10-T_0_, had Cry1Ab/1Ac expression with the expected molecular size at about 68 kDa; the remaining T_0_ plants either did not show any expression of the Cry1Ab/1Ac protein or expressed truncated Cry1Ab/1Ac proteins (data not shown). Transgenic L10-T_0_ was selected for further study, while the other undesirable T_0_ plants were discarded. L10-T_0_ had five copies of T-DNA detected by southern blot analysis using the restriction enzyme *Nco*I, which has cutting sites upstream only of *P*_*Pepc*_*:Cry1Ab/1Ac:T*_*Nos*_, and the *Cry1Ab/1Ac* gene probe (Figure 2A). The above-mentioned PCR analysis also indicates that L10-T_0_ contains both marker-free and marker-containing T-DNAs (Table 1). We then performed genetic analysis in order to obtain functional and marker-free transgenic T_1_ plants that carry the *P*_*Pepc*_*:Cry1Ab/1Ac:T*_*Nos*_ gene only. Twenty T_1_ progeny of L10-T_0_ were obtained and they were screened by PCR for individuals that contained only marker-free T-DNA (Table 2). Three T_1_ plants, L10-T_1_-5, L10-T_1_-10 and L10-T_1_-18, showed PCR amplification of the *Cry1Ab/1Ac* gene and the P1-P4 fragment, but not for P1-P2 and P3-P4 fragment (Table 2 and Figure 3; data for L10-T_1_-5 are not shown). The results indicate that the three T_1_ plants have inherited the marker-free T-DNA from L10-T_0_. L10-T_1_-10 and L10-T_1_-18 were selected for further analysis, whereas L10-T_1_-5 died from pathogen infection. Southern blot analysis indicated that both L10-T_1_-10 and L10-T_1_-18 carry one copy of T-DNA, which is the marker-free T-DNA identified by PCR analysis (Figure 2B and C). More importantly, the expected 3.5-kb *Sph*I-*Kpn*I fragment was detected in L10-T_1_-10 and L10-T_1_-18 by southern blot analysis using restriction enzymes *Sph*I and *Kpn*I and the *Cry1Ab/1Ac* probe (Figure 2D), indicating that the two marker-free transgenic plants contain the intact *P*_*Pepc*_*:Cry1Ab/1Ac:T*_*Nos*_ gene. For both PCR and southern blot analyses, no signal was detected with non-transgenic MD44 plants (Table 2, Figure 2 and Figure 3).

**Table 2:** **PCR analysis of the L10 T**_**1 **_**transgenic plants**

**T**_**1 **_**plant**	**P1-P2**^**1**^	**P3-P4**^**2**^	**P1-P4**^**3**^	***Hpt***^**4**^	***Cry1Ab/1Ac***^**5**^
pCCreloxPBt^6^	+	+	-	+	+
MD44	-	-	-	-	-
L10-T_1_-1	-	+	-	+	+
L10-T_1_-2	-	-	+	+	+
L10-T_1_-3	-	+	-	+	+
L10-T_1_-4	-	+	+	+	+
L10-T_1_-5	-	-	+	-	+
L10-T_1_-6	-	+	-	+	+
L10-T_1_-7	-	+	-	+	+
L10-T_1_-8	-	+	-	+	+
L10-T_1_-9	-	+	-	+	+
L10-T_1_-10	-	-	+	-	+
L10-T_1_-11	-	+	+	+	+
L10-T_1_-12	-	+	-	+	+
L10-T_1_-13	-	+	-	+	+
L10-T_1_-14	-	-	-	-	-
L10-T_1_-15	-	+	+	+	+
L10-T_1_-16	-	+	+	+	+
L10-T_1_-17	-	+	+	+	+
L10-T_1_-18	-	-	+	-	+
L10-T_1_-19	-	+	+	+	+
L10-T_1_-20	-	+	+	+	+

^1^The primers for detecting the presence of P1-P2 fragment were P1 and P2 [34]; ^2^the primers for detecting the presence of P3-P4 fragment were P3 and P4 [34]; ^3^the primers for detecting the excision of the *lox*P fragment or the presence of P1-P4 fragment were P1 and P4 [34]; ^4^the primers for detecting the presence of the *Hpt* gene were Hpt F and Hpt910-1; ^5^the primers for detecting the presence of the *Cry1Ab/1Ac* gene were Bt F1 and Bt R1; ^6^binary construct used for transgenic *J. curcas* production [34].

### Expression of the *P*_*Pepc*_*:Cry1Ab/1Ac:T*_*Nos*_ gene in marker-free plants L10-T_1_-10 and L10-T_1_-18

**Figure 4:**
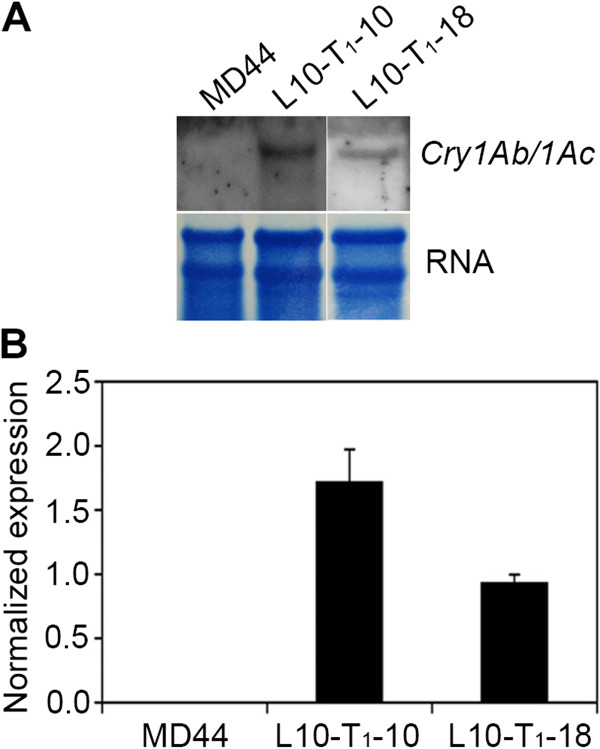
**Expression of the *Cry1Ab/1Ac *gene in transgenic *J. curcas *plants. (A)** Detection of *Cry1Ab/1Ac* gene transcripts by northern blot analysis. The upper panel shows the result of northern blot analysis detected by a *Cry1Ab/1Ac* probe. The lower panel is the image of RNA loading assessed by methylene blue staining. MD44, *J. curcas* cultivar MD44; L10-T_1_-10 and L10-T_1_-18, transgenic T_1_ plants. **(B)** Detection of *Cry1Ab/1Ac* gene transcripts by qRT-PCR. Results are shown as the normalized expression with the expression level of the *Cry1Ab/1Ac* gene in L10-T_1_-18 set as 1. The experiments were performed in triplicate and the data are presented as means ± SD.

**Figure 5:**
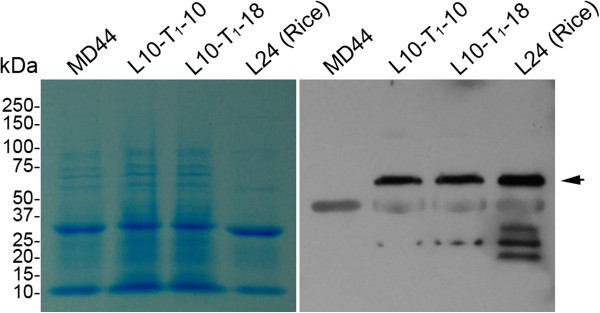
**Detection of Cry1Ab/1Ac proteins in transgenic *J. curcas *plants.** The Cry1Ab/1Ac proteins were detected with anti-CRY1Ab polyclonal antibodies. The Cry1Ab/1Ac proteins expressed in L24 of transgenic rice were used as the positive control. The proteins stained with Coomassie blue in duplicate SDS-PAGE gel serve as protein loading controls (*left panel*). Arrow indicates the position of Cry1Ab/1Ac. MD44, *J. curcas* cultivar MD44; L10-T_1_-10 and L10-T_1_-18, transgenic T_1_ plants; L24 (Rice), transgenic rice carrying the *P*_*Pepc*_*:Cry1Ab/1Ac:T*_*Nos*_ gene [34].

The transcripts of the *P*_*Pepc*_*:Cry1Ab/1Ac:T*_*Nos*_ gene in L10-T_1_-10 and L10-T_1_-18 were detected by northern blot analysis as well as by real-time quantitative reverse transcription PCR (qRT-PCR) (Figure 4A and B). No signal was detected in non-transgenic MD44 plants in either of the two experiments (Figure 4A and B). The Cry1Ab/1Ac proteins expressed in transgenic *J. curcas* plants were detected by western blot analysis and anti-CRY1Ab polyclonal antibodies. The Cry1Ab/Ac proteins were expressed in L10-T_1_-10 and L10-T_1_-18, which had similar molecular size to that of Cry1Ab/1Ac expressed in rice (Figure 5). No Cry1Ab/1Ac signal was detected in non-transgenic MD44 plants (Figure 5). However, a non-specific band with molecular size at about 50 kDa was detected by anti-CRY1Ab polyclonal antibodies in both MD44 and transgenic *J. curcas* plants (Figure 5). The results demonstrated that the *P*_*Pepc*_*:Cry1Ab/Ac:T*_*Nos*_ gene expresses normally in leaf tissues of transgenic *J. curcas* plants.

### Cry1Ab/1Ac expressed in *J. curcas* provides resistance to *A. micaceanus*

**Figure 6:**
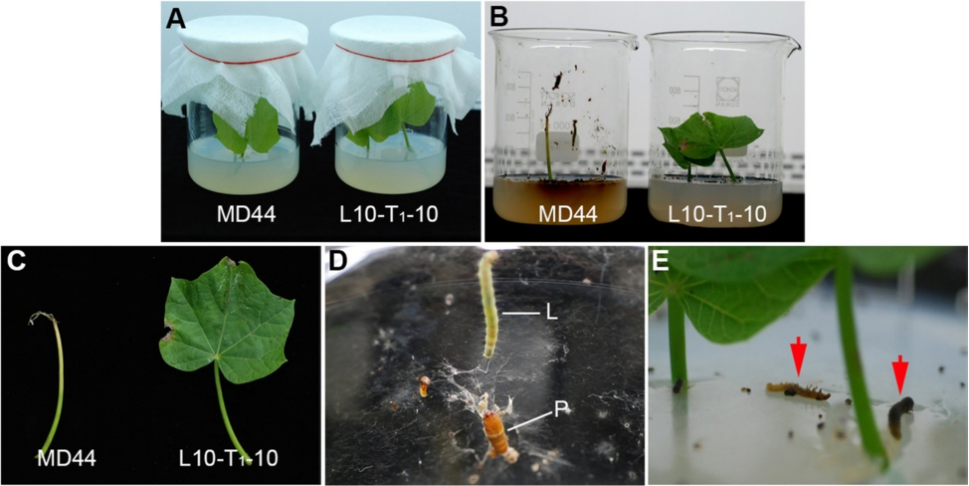
**Bioassay on insecticidal activity of Cry1Ab/1Ac in transgenic *J. curcas *plants against *A. micaceanus. *(A, B)** Leaf explants from MD44 and transgenic *Cry1Ab/1Ac* plant L10-T_1_-10 artificially infested with the second to third instar larvae of *A. micaceanus* in beakers. Photos were taken at 0 day **(A)** and 4 days **(B)** after infestation, respectively. **(C)** Feeding damage on leaves of MD44 and L10-T_1_-10 by *A. micaceanus* larvae. Photos were taken at 4 days after infestation. **(D)** Live larva and pupa of *A. micaceanus* after feeding on leaves of MD44 at 4 days after infestation. L, Larva; P, pupa. **(E)** Death of larvae of *A. micaceanus* after feeding on leaves of L10-T_1_-10 at 4 days after infestation. Arrows indicate two dead larvae.

*A. micaceanus* larvae, at the second to third instar stages, were collected from *J. curcas* plants grown in an in-house farm and used for insecticidal bioassay. The larvae were fed with leaf explants from MD44 or L10-T_1_-10 (Figure 6A). After 4 days of infestation, the larvae feeding on non-transgenic MD44 completely ate up the two leaf explants in each beaker, whereas the larvae feeding on L10-T_1_-10 caused little damage to the leaf explants (Figure 6B and C). In total, 14 of the 15 *A. micaceanus* larvae feeding on MD44 in the three repeated experiments remained alive and healthy, and one larva in experiment I transformed into a pupa, which might be due to the third instar larvae used for this experiment (Figure 6D; Table 3). On the contrary, 14 of the 15 *A. micaceanus* larvae feeding on L10-T_1_-10 died at 2 to 3 days after infestation (Figure 6E; Table 3). Only one larva survived and transformed into a pupa after feeding on L10-T_1_-10 in experiment I (Table 3). The larvae mortality in explants of transgenic *J. curcas* ranged from 80 to 100% in the three repeated experiments (Table 3). The bioassay results clearly demonstrated that the Cry1Ab/1Ac proteins expressed in transgenic *J. curcas* have strong insecticide activity against *A. micaceanus* larvae.

**Table 3:** **Bioassay using artificial infestation of *A. micaceanus *larvae on leaf explants of MD44 and transgenic plant L10-T**_**1**_**-10**

**Plant**	**Larvae upon infestation, number**	**Larvae or pupa after infestation, number**	**Mortality %**^**1**^
**Experiment I**			
MD44	5	4 larvae + 1 pupa	0
L10-T_1_-10	5	1 pupa	80%
**Experiment II**			
MD44	5	5 larvae	0
L10-T_1_-10	5	0	100%
**Experiment III**			
MD44	5	5 larvae	0
L10-T_1_-10	5	0	100%

^1^Viability and mortality of insects were scored at 4 days after infestation.

## Discussion

Using *Agrobacterium*-mediated transformation and a chemically regulated, Cre/*loxP*-mediated DNA recombination system, we have obtained one transgenic *J. curcas* line that contains a single copy of the *P*_*Pepc*_*:Cry1Ab/1Ac:T*_*Nos*_ gene within a marker-free T-DNA. Twenty T_0_ plants were produced in this study but one marker-free line was obtained. The efficiency of obtaining a marker-free transgenic line was only 5%. The major reason for the low efficiency in obtaining marker-free transgenic line may be due to the inefficient excision of the *loxP* fragment in the T-DNA after *β*-estradiol induction, which occurred in 7 of the 20 T_0_ plants obtained (Table 1). In this study, the *β*-estradiol induction was applied on the hygromycin-resistant shoots rather than on the hygromycin-resistant calli in order to obtain as many transgenic plants as possible. In this case, the *β*-estradiol might not be able to uniformly and efficiently access all types of cells, especially the germline cells in the regenerated shoots. Further study may be required to test the introduction of *β*-estradiol induction on the hygromycin-resistant calli rather than the hygromycin-resistant shoots. Another reason could be the result of incomplete *loxP* fragment excision from T-DNA in transgenic plants that contain multiple copies of T-DNA. In this study, the two marker-free T_1_ plants, L10-T_1_-10 and L10-T_1_-18, were obtained from T_1_ progeny of L10-T_0_, which contained at least five copies of T-DNA. Finally, truncated T-DNA integration containing only one *loxP* site may make it impossible to perform Cre/*loxP*-mediated DNA recombination, which further reduces the efficiency of obtaining marker-free transgenic plants. Incomplete *loxP* fragment excision may be unavoidable due to the common presence of multiple T-DNA insertion and truncated T-DNA integration [34,36,37]. Nevertheless, the marker-free transgenic *J. curcas* plants generated in this study reduce the biosafety concerns of the marker gene on the environments.

Due to the presence of toxins such as phorbol ester and curcin in seeds, the storage lipids from *J. curcas* seeds are mainly used as biofuel or in other industrial applications rather than as human food or animal feed. The *Bt δ*-endotoxin, as an effective biological insecticide, has been successfully used for generation of transgenic plants for insect resistance in both crops and trees. Therefore, the utilization of the *cry* gene in developing transgenic *J. curcas* for insect resistance is an ideal method for insect control and will unlikely give rise to biosafety concern in the food chain of human beings. In this study, the Cry1Ab/1Ac proteins produced in transgenic *J.curcas* plants had strong insecticidal activity to *A. micaceanus* larvae. Previously, the hybrid Cry protein expressed in transgenic rice showed strong larvicidal activity that kills lepidopteran pests, the most serious of which include the yellow stem borer (*S. incertulas*), the striped stem borer (*Chilo suppressalis*) and rice leaffolder (*C. medinalis*) [33,34,38]. The Cry proteins from *Bt* have been found to show specifically toxic activity against numerous insect species of the orders Lepidoptera, Diptera, Coleoptera, Hymenoptera and nematodes [1]. It was reported that there are six families of Coleoptera and three families of Lepidoptera that can attack *J. curcas* plants [20]. Therefore, in the future it is worth testing whether the transgenic *J. curcas* generated in this study can provide insecticidal activity for these insects of *J. curcas*.

## Conclusion

We have produced one marker-free transgenic *J. curcas* line that carries a single copy of the *P*_*Pepc*_*:Cry1Ab/1Ac:T*_*Nos*_ gene. The Cry1Ab/1Ac proteins expressed in the transgenic *J. curcas* line provide high resistance to *A. micaceanus* larvae. The marker-free transgenic line, designed as L10 for further study, can be used for *J. curcas* breeding for insect resistance to *A. micaceanus* and probably for other *Bt δ*-endotoxin-sensitive insects on *J. curcas* plants.

## Materials and methods

### Plant materials and growth conditions

*J. curcas* L. cv. MD44 was used for *J. curcas* transformation. MD44 and transgenic *J. curcas* plants were grown in the greenhouse under natural climate conditions at a temperature of 30 to 32°C for 12.5 h (light) and 24 to 26°C for 11.5 h (dark).

### Plant transformation

The binary construct pCCreloxPBt [34] was used for MD44 transformation. pCCreloxPBt carries a *P*_*Pepc*_*:Cry1Ab/1Ac:T*_*Nos*_ gene and a chemically regulated Cre/*loxP* system for the excision of the selection marker gene [34]. Agrobacterium-mediated transformation of *J. curcas* was carried out according to the method described previously [35]. In brief, the cotyledon discs at the size of 0.3 × 0.3 cm^2^ were co-cultivated with *Agrobacterium tumefaciens* strains AGL1 harboring pCCreloxPBt on co-cultivation medium [39] for 2 to 3 days at 22°C in darkness. The co-cultivated cotyledon discs were rinsed thoroughly with sterile water and then with suspension medium containing 300 mg/L cefotaxime [39]. Cotyledon discs were cultured on callus formation medium [39] at 25°C in darkness for 3 weeks. The cotyledon discs carrying newly emerged hygromycin-resistant calli were transferred onto shoot regeneration medium I [39] and cultured for 3 weeks at 25°C under 16-h light/8-h dark cycles. The regenerated shoots were sub-cultured on shoot regeneration medium II [39]. The hygromycin-resistant shoots at about 2 to 3 mm were transferred onto *β*-estradiol induction medium without hygromycin [39] to induce marker excision. After 2 weeks, the *β*-estradiol-treated shoots were transferred back to the shoot regeneration medium II without hygromycin [39]. After 4 weeks, the regenerated shoots were transferred onto shoot elongation medium [39] for elongation and bud multiplication. The elongated shoots at about 2.5-cm length were rooted on rooting medium [39]. The putative transgenic plants with a healthy root system were eventually transplanted into soil-filled pots in the greenhouse.

### PCR analysis

PCR analysis for the presence of transgenes as well as Cre/*loxP*-mediated DNA recombination in transgenic plants was carried out according to the methods described previously [34]. The PCR primers used in this study are listed in Table 4.

**Table 4:** DNA primers used in this study

**Primer**	**Nucleotide sequence (5′–3′)**	**Reference**
P1	GAATTGTCGAGGTCGAAGATC	[34]
P2	ATAGTGAAACAGGGGCAATGG	[34]
P3	ACGGCGAGTTCTGTTAGGTC	[34]
P4	GAAGATACACGGATTGAGGAGAG	[34]
Hpt F	AAAAAGCCTGAACTCACCGCGACGT	This study
Hpt910-1	TACTTCTACACAGCCATCGGTCCA	This study
Bt F1	AGGCCATACAACTGCTTGAG	This study
Bt R1	CTGTAGACACCCTGACCTAG	This study
Bt F2	TCATCCATCTTCTCCAATACAG	This study
Bt R2	GTAACTGGAATGAACTCGAATC	This study
JcActin F1	TAATGGTCCCTCTGGATGTG	This study
JcActin R1	AGAAAAGAAAAGAAAAAAGCAGC	This study

### Southern blot analysis

*J. curcas* genomic DNA was isolated from leaf tissues as described previously [40]. About 2 μg of DNA was digested with proper restriction enzymes, separated on 0.8% agarose gel and then blotted to Hybond™-N^+^ nylon membrane (Amersham Biosciences, Little Chalfont, Buckinghamshire, UK). Southern blots were hybridized with DIG-labelled DNA probes for *Cry1Ab/1Ac* and hygromycin phosphotransferase gene (*Hpt*), respectively, according to standard procedures. The primer pairs for amplification of DNA probes were Bt F1/Bt R1 for the *Cry1Ab/1Ac* gene and Hpt F/Hpt910-1 for the *Hpt* gene, respectively (Table 4).

### Northern blot analysis

Total RNA was isolated from leaf tissues of *J. curcas* using the methods described previously [40]. About 10 μg total RNA was fractionated on a 1.2% formaldehyde agarose gel and blotted onto a Hybond™ N^+^ membrane (Amersham Biosciences). The northern blot hybridization and the labeling of the *Cry1Ab/1Ac* gene probe were similar to the methods described for the southern blot analysis.

### Real-time quantitative reverse transcription PCR (qRT-PCR)

qRT-PCR was carried out according to the method described previously with minor modification [41]. The first strand cDNA was synthesized using iScript cDNA synthesis kit (Bio-Rad, Hercules, CA, USA). PCR reaction (15 μl) was conducted on a CFX96 real-time system containing 2 μl first strand cDNA templates, 1 × SsoFast EvaGreen supermix (Bio-Rad) and 500 nM forward primer Bt F2 and reverse primer Bt R2 (Table 4). The actin gene 1 (*JcActin1*) of *J. curcas* was used as control. The primer pair for the *Cry1Ab/1Ac* gene was Bt F2/Bt R2, and the primer pair for the *JcActin1* gene was JcActin F1/JcActin R1 (Table 4).

### Western blot analysis

Total proteins were extracted from *J. curcas* leaves with a homogenization buffer (0.1 M Tris-HCl, pH8.0, 0.01 M MgCl_2_, 18% (w/v) sucrose, 40 mM *β*-mercaptoethanol). Total protein concentration was determined with the Bradford method [42]. About 10 μg of each protein sample was separated on an 8% SDS-PAGE, followed by blotting onto polyvinylidene fluoride (PVDF) membranes (Bio-Rad). The Cry1Ab/1Ac proteins from the rice line L24, a marker-free transgenic line carrying the *P*_*Pepc*_*:Cry1Ab/1Ac:T*_*Nos*_ gene [34], served as the positive control. Cry1Ab/1Ac proteins were detected with anti-CRY1Ab polyclonal antibodies (Abcam, Cambridge, UK) and horseradish peroxidase-coupled secondary antibodies (Bio-Rad).

### Insect bioassay

A beaker method was utilized to check if the Cry1Ab/1Ac proteins expressed in the transgenic *J. curcas* plants had insecticidal activity towards *A. micaceanus* larvae. Leaf explants from transgenic *J. curcas* plants or non-transgenic MD44 plants were sterilized and put on 1% Agar medium in beakers. Five, second to third instar *A. micaceanus* larvae, collected from *J. curcas* plants grown in an inhouse farm were fed onto *J. curcas* leaves. The beakers were sealed with cheese cloth to prevent the larvae from escaping. The feeding assay was conducted in a growth chamber at a temperature of 28°C, relative humidity of 80% and photoperiod of 12 h. Damage on leaf tissues and the larval mortality were observed and photographed at 4 days after infestation. The experiment was repeated three times and the results are representative of each independently conducted experiment.

## Abbreviations

bp: base pairs; Bt: *Bacillus thuringiensis*; PEPC: phosphoenolpyruvate carboxylase; Cry: Crystalline; qRT-PCR: real-time quantitative reverse transcription PCR.

## Competing interests

The authors declare no competing financial interests.

## Authors’ contributions

YZ and GK designed the experiments. GK and MH conducted the experiments. GK and YZ analyzed the data and wrote the article. All the authors have read and approved the final manuscript.

## Authors’ information

GK is a Research Fellow, MH is a Senior Research Officer and YZ is the Head of the laboratory and an Associate Director of the Institute’s Strategic Research Program at the Temasek Life Sciences Laboratory, 1 Research Link, NUS, Singapore 117604 and an Adjunct Assistant Professor of the Department of Biological Sciences, 14 Science Drive, National University of Singapore, Singapore 117543, Republic of Singapore.

## References

[B1] Schnepf E Crickmore N Van Rie J Lereclus D Baum J Feitelson J Zeigler D Dean D Bacillus thuringiensis and its pesticidal crystal proteins Microbiol Mol Biol Rev 1998 62 775 806 9729609 10.1128/mmbr.62.3.775-806.1998 PMC98934

[B2] Tojo A Aizawa K Dissolution and degradation of Bacillus thuringiensis δ-endotoxin by gut juice protease of the silkworm Bombyx mori Appl Environ Microbiol 1983 45 576 580 16346206 10.1128/aem.45.2.576-580.1983 PMC242326

[B3] Milne R Kaplan H Purification and characterization of a trypsin-like digestive enzyme from spruce budworm (*Chroristoneura fumiferana*) responsible for the activation of δ-endotoxin from *Bacillus thuringiensis* Insect Biochem Mol Biol 1993 23 663 673 10.1016/0965-1748(93)90040-Y 8353523

[B4] Knowles BH Dow JA The crystal δ‒endotoxins of Bacillus thuringiensis: Models for their mechanism of action on the insect gut Bioessays 1993 15 469 476 10.1002/bies.950150706

[B5] Sacchi VF Parenti P Hanozet GM Giordana B Lüthy P Wolfersberger MG *Bacillus thuringiensis* toxin inhibits K^+^-gradient-dependent amino acid transport across the brush border membrane of *Pieris brassicae* midgut cells FEBS Lett 1986 204 213 218 10.1016/0014-5793(86)80814-6

[B6] Wolfersberger MG Neither barium nor calcium prevents the inhibition by *Bacillus thuringiensis* δ-endotoxin of sodium or potassium gradient dependent amino acid accumulation by tobacco hornworm midgut brush border membrane vesicles Arch Insect Biochem Physiol 1989 12 267 277 10.1002/arch.940120406

[B7] Barton KA Whiteley H Yang N-S *Bacillus thuringiensis* delta-endotoxin expressed in transgenic *Nicotiana tabacum* provides resistance to lepidopteran insects Plant Physiol 1987 85 1103 1109 10.1104/pp.85.4.1103 16665812 PMC1054402

[B8] Vaeck M Reynaerts A Höfte H Jansens S De Beuckeleer M Dean C Zabeau M Montagu MV Leemans J Transgenic plants protected from insect attack Nature 1987 328 33 37 10.1038/328033a0

[B9] Kumar S Chandra A Pandey K *Bacillus thuringiensis* (*Bt*) transgenic crop: an environment friendly insect-pest management strategy J Environ Biol 2008 29 641 653 19295059

[B10] Kleiner K Ellis D McCown B Raffa K Field evaluation of transgenic poplar expressing a *Bacillus thuringiensis* cry1A(a) δ-endotoxin gene against forest tent caterpillar (Lepidoptera: Lasiocampidae) and gypsy moth (Lepidoptera: Lymantriidae) following winter dormancy Environ Entomol 1995 24 1358 1364

[B11] McCown B McCabe D Russell D Robison D Barton K Raffa K Stable transformation of Populus and incorporation of pest resistance by electric discharge particle acceleration Plant Cell Rep 1991 9 590 594 24220719 10.1007/BF00232339

[B12] Dandekar AM McGranahan GH Vail PV Uratsu SL Leslie C Tebbets JS Low levels of expression of wild type *Bacillus thuringiensis* var. *kurstaki* cry1A(c) sequences in transgenic walnut somatic embryos Plant Sci 1994 96 151 162 10.1016/0168-9452(94)90232-1

[B13] Shin D-I Podila GK Huang Y Karnosky DF Transgenic larch expressing genes for herbicide and insect resistance Can J Forest Res 1994 24 2059 2067 10.1139/x94-264

[B14] Ellis D McCabe D McInnis S Ramachandran R Russell D Wallace K Martinell B Roberts D Raffa K McCown B Stable transformation of Picea glauca by particle acceleration Bio/Technology 1993 11 84 89 10.1038/nbt0193-84

[B15] Peña L Séguin A Recent advances in the genetic transformation of trees Trends Biotechnol 2001 19 500 506 10.1016/S0167-7799(01)01815-7 11711193

[B16] Tang W Tian Y Transgenic loblolly pine (*Pinus taeda* L.) plants expressing a modified δ-endotoxin gene of *Bacillus thuringiensis* with enhanced resistance to *Dendrolimus punctatus* Walker and *Crypyothelea formosicola* Staud J Exp Bot 2003 54 835 844 10.1093/jxb/erg071 12554726

[B17] Harcourt R Kyozuka J Floyd R Bateman K Tanaka H Decroocq V Llewellyn D Zhu X Peacock W Dennis E Insect-and herbicide-resistant transgenic eucalyptus Mol Breed 2000 6 307 315 10.1023/A:1009676214328

[B18] Génissel A Leplé J-C Millet N Augustin S Jouanin L Pilate G High tolerance against *Chrysomela tremulae* of transgenic poplar plants expressing a synthetic cry3Aa gene from *Bacillus thuringiensis* ssp *tenebrionis* Mol Breed 2003 11 103 110 10.1023/A:1022453220496

[B19] Gubitz GM Mittelbach M Trabi M Exploitation of the tropical oil seed plant Jatropha curcas L Bioresour Technol 1999 67 73 82 10.1016/S0960-8524(99)00069-3

[B20] Datinon B Glitho A Tamò M Amevoin K Goergen G Kpindou O Inventory of major insects of *Jatropha curcas* L.(Euphorbiaceae) and their natural enemies in Southern Benin J Agric Biol Sci 2013 8 711 718

[B21] Grimm C Evaluation of damage to physic nut (Jatropha curcas) by true bugs Entomol Exp Appl 1999 92 127 136 10.1046/j.1570-7458.1999.00532.x

[B22] Prabhakar M Prasad Y Rao G Venkateswarlu B A new record of longicorn beetle, Acanthophorus rugiceps, from India as a root borer on physic nut, Jatropha curcas, with a description of life stages, biology, and seasonal dynamics J Insect Sci 2012 12 141 23461741 10.1673/031.012.14101 PMC3648335

[B23] Rodrigues SR Oliveira HN Santos W Abot AR Biological aspects and damage of Pachycoris torridus on physic nut plants Bragantia 2011 70 356 360 10.1590/S0006-87052011000200015

[B24] Shanker C Dhyani S Insect pests of Jatropha curcas L. and the potential for their management Curr Sci 2006 91 162 163

[B25] Srinivasa Rao C Pavani Kumari M Wani SP Marimuthu S Occurrence of black rot in Jatropha curcas L. plantations in India caused by Botryosphaeria dothidea Curr Sci 2011 100 1547 1549

[B26] Gao S Qu J Chua N-H Ye J A new strain of Indian cassava mosaic virus causes a mosaic disease in the biodiesel crop Jatropha curcas Arch Virol 2010 155 607 612 10.1007/s00705-010-0625-0 20224893

[B27] Alford DV A textbook of agricultural entomology 1999 1 Blackwell Science Ltd

[B28] Waterhouse D The major arthropod pests and weeds of agriculture in Southeast Asia: distribution, importance and origin 1993 Canberra, Australia: Australian Centre for International Agricultural Research

[B29] Rianti P Suryobroto B Atmowidi T Diversity and effectiveness of insect pollinators of *Jatropha curcas* L. (Euphorbiaceae) HAYATIJ Biosci 2010 17 38 10.4308/hjb.17.1.38

[B30] Vakenti J Campbell C Madsen H A strain of fruittree leafroller, Archips argyrospilus (Lepidoptera: Tortricidae), tolerant to azinphos-methyl in an apple orchard region of the Okanagan Valley of British Columbia Can Entomologist 1984 116 69 73 10.4039/Ent11669-1

[B31] Sorensen A Falcon L Comparison of microdroplet and high volume application of Bacillus thuringiensis on pear: suppression of fruit tree leafroller (Archips argyrospilus) and coverage on foliage and fruit Environ Entomol 1980 9 350 358

[B32] AliNiazee M Evaluation of Bacillus thuringiensis against Archips rosanus (Lepidoptera: Tortricidae) Can Entomologist 1974 106 393 398 10.4039/Ent106393-4

[B33] Tu J Zhang G Datta K Xu C He Y Zhang Q Khush GS Datta SK Field performance of transgenic elite commercial hybrid rice expressing *Bacillus thuringiensis* delta-endotoxin Nat Biotechnol 2000 18 1101 1104 10.1038/80310 11017051

[B34] Qiu C Sangha JS Song F Zhou Z Yin A Gu K Tian D Yang J Yin Z Production of marker-free transgenic rice expressing tissue-specific *Bt* gene Plant Cell Rep 2010 29 1097 1107 10.1007/s00299-010-0893-x 20593185

[B35] Qu J Mao H-Z Chen W Gao S-Q Bai Y-N Sun Y-W Geng Y-F Ye J Development of marker-free transgenic Jatropha plants with increased levels of seed oleic acid Biotechnol Biofuels 2012 5 10 10.1186/1754-6834-5-10 22377043 PMC3316142

[B36] Sreekala C Wu L Gu K Wang D Tian D Yin Z Excision of a selectable marker in transgenic rice (Oryza sativa L.) using a chemically regulated Cre/loxP system Plant Cell Rep 2005 24 86 94 10.1007/s00299-004-0909-5 15662501

[B37] Yin Z Wang G-L Evidence of multiple complex patterns of T-DNA integration into the rice genome Theor Appl Genet 2000 100 461 470 10.1007/s001220050060

[B38] Wang Y Hu H Huang J Li J Liu B Zhang G Determination of the movement and persistence of Cry1Ab/1Ac protein released from *Bt* transgenic rice under field and hydroponic conditions Soil Biol Biochem 2013 58 107 114

[B39] Mao H Ye J Chua N Genetic transformation of *Jatropha curcas* International Application No: PCT/SG2009/000479 USA 2009

[B40] Gu K Chiam H Tian D Yin Z Molecular cloning and expression of heteromeric ACCase subunit genes from Jatropha curcas Plant Sci 2011 180 642 649 10.1016/j.plantsci.2011.01.007 21421413

[B41] Gu K Yi C Tian D Sangha JS Hong Y Yin Z Expression of fatty acid and lipid biosynthetic genes in developing endosperm of Jatropha curcas Biotechnol Biofuels 2012 5 1 15 10.1186/1754-6834-5-1 22809288 PMC3457857

[B42] Bradford MM A rapid and sensitive method for the quantitation of microgram quantities of protein utilizing the principle of protein-dye binding Anal Biochem 1976 72 248 254 10.1016/0003-2697(76)90527-3 942051

